# Dynamical Systems Theory in Psychology: Assistance for the Lay Reader is Required

**DOI:** 10.3389/fpsyg.2012.00382

**Published:** 2012-10-04

**Authors:** Lois A. Gelfand, Sally Engelhart

**Affiliations:** ^1^Department of Psychology, University of PennsylvaniaPhiladelphia, PA, USA

Dynamical Systems Theory (DST) has generated interest and excitement in psychological research, as demonstrated by the recent statement, “…the dynamical perspective has emerged as a primary paradigm for the investigation of psychological processes at different levels of personal and social reality” (Vallacher et al., 2010, p. 263).

What is less clear to the authors is the degree to which this excitement is justified. Like many psychology researchers, we were initially unfamiliar with the concepts, terminology, and techniques used in DST modeling (an approach that was developed in physics), which made it difficult to judge applications of DST in the articles we encountered. After reading introductory DST material, we developed some opinions about how authors of DST-related articles could help psychologists who are not familiar with DST [hereafter referred to as “lay reader(s)”] to begin to make judgments about their work. In order for DST to be a useful methodology for psychology research, we believe, DST-based work must be reasonably accessible to other psychologists.

Although we are restricting our discussion to the application of DST methodology to clinical psychology research, we believe that the following three recommendations may be applied to the field of psychology more broadly.

## Maintain a Distinction between Dynamical and Non-Dynamical Models

A defining feature of a dynamical model is that the values of the variables in a dynamical system at one time are modeled as functions of those same variables at earlier times. One characteristic, therefore, that distinguishes dynamical models from the statistical models commonly applied in clinical psychology research is that in dynamical models the same variables serve, in a sense, as both dependent and independent variables. Another way of saying this is that dynamical systems are, by definition, feedback models.

For example, *X*(*t* + 1) = *aX*(*t*) constitutes an extremely simple dynamical system with one variable (*X*) and a constant (the coefficient *a*) that, multiplied by *X* at time *t*, defines *X* at time *t* + 1.

In contrast, models in which dependent variables are distinct from independent variables, such as OLS regression and hierarchical linear modeling (HLM, which can also be used to perform non-linear growth curve analyses), are not feedback models, and thus are not dynamical systems.

In a 1994 review article, Barton seemed to blur this distinction, implying that all statistical models are dynamical and differ primarily in whether they involve linear or non-linear equations.

From a mathematical perspective, dynamics can be thought of as linear or non-linear… Linear equations… are… the cornerstone of statistics. When we perform an analysis of variance or enter data into a multiple regression equation, we are using linear equations to describe the relationships among variables (pp. 5–6).

In a response to Barton (1994), Mandel (1995, 107) clarified the distinction between “dynamical and static approaches (as well as linear and non-linear models),”such that OLS regression, for example, would be considered “linear static” and non-linear growth curve analysis via HLM would be considered “non-linear static.” However, this distinction is not always clearly maintained in the psychology literature.

For example, Hayes et al. (2007) related their study, in which they examined non-linear trajectories of depression change during treatment, to DST, although there were no dynamical components to their model. That is, their dependent and independent variables were distinct (i.e., no feedback), and their data analyses were conducted via “static” approaches (non-linear growth curve analysis via HLM). Nonetheless, they described the focus of their study using DST terminology (e.g., “critical fluctuations,” p. 410). By doing so, they may have led lay readers to conclude, incorrectly, that the trajectories of depression change they reported fit into a DST framework, that their analyses constituted applications of DST theory and methods to clinical psychology, and that judgments of the presented research would be relevant to judging the usefulness of DST in clinical psychology research.

To maintain the distinction between dynamical and non-dynamical models, researchers reporting on non-dynamical models can simply omit any reference to DST. Researchers presenting non-dynamical models who choose to refer to DST terminology should explicitly state that their models are not dynamical, and, furthermore, should make clear what the relevance of DST is to the presented research. For example, are DST concepts being presented metaphorically? Do the researchers speculate that a dynamical process underlies their data, but refrain from examining a dynamical model? If so, what evidence supports the speculation, and why is a dynamical model not investigated? Maintaining a clear distinction between dynamical and non-dynamical models will assist the lay reader in making judgments about the usefulness of DST in clinical psychology research.

## Maintain a Clear Distinction between Influences on the Variables from the Proposed Model and Other Influences.

DST, by its nature, involves the study of processes that unfold over time in a deterministic manner (absent any perturbations), from an initial state, based solely on the functional relationships among the variables in the system. In the context of clinical psychology, it may be difficult to identify variables that operate in such a deterministic manner or to construct models that adequately characterize their interactions. Difficulties may arise from a number of sources, including the intentional actions of participants and the difficulties in isolating psychological variables from the myriad environmental influences that affect human beings. Unless DST researchers explicitly state otherwise, lay readers may assume that any influences mentioned by the researchers originate from the proposed model, and thus be unable to accurately assess the usefulness of the model. Therefore, when researchers discuss the influences on variables they examine, we believe that it is incumbent on them to be particularly careful in distinguishing between those influences arising from the proposed model and other influences.

For example, Peluso et al. (2012, 51), presented non-linear dynamical models of the changes over time of psychotherapist and client emotional valences, in which each participant’s emotional valence was modeled as deterministic functions of both the other participant’s emotional valence and their own emotional valence at the immediately preceding time.

The authors also suggested that psychotherapists be “mindful of” and “monitor” these valences and how strongly they impact one another, presumably with the idea that the psychotherapists would adjust their own values in order to improve therapy outcomes. Implicit, then, was an assumption that there were two sources of influence on psychotherapist emotional valence – one from a dynamical system, in which the emotional valence changes deterministically, and the other from outside of the dynamical system, involving direct volitional changes. However, because the authors did not make this distinction explicit, the lay reader may assume, incorrectly, that volitional changes in variables are consistent with their model, when in fact they are inconsistent both with the specific model and also with the deterministic framework of DST more generally.

In a different type of example, Chow et al. (2005) used a linear dynamical model to describe periodic fluctuations in hedonic level. Empirical results showed a weekly periodicity in hedonic level; specifically, the undergraduate subjects in the study, who were studied in their natural environment, enjoyed themselves more on weekends than on weekdays.

In this case, the hypothesized dynamical model posited was a simple model in which hedonic level at one time varied only in relation to hedonic level at a previous time. However, the fact that the hedonic cycle appeared to be entrained to a weekly calendar cycle suggests that other influences may have been at work (i.e., behavioral demands are different on weekdays versus weekends for students). Because Chow et al. (2005) did not explicitly state that the entrainment of periodic hedonic fluctuations to an external environmental (calendar) cycle was not part of their model, the lay reader may assume, incorrectly, that it was.

When researchers present DST-related work, it might be helpful for them to include two separate sections in the discussion: one for influences on the variables that arise from the proposed model; and another for other influences. This would put lay readers in a better position to judge the usefulness of the model, and thus to better evaluate the role of DST in psychology research.

## Include a Time Series Plot from the Empirical Data or the Model, and if Both are Available, Show Them Together

Dynamical systems involve changes in variables that unfold over time. Although the graphical techniques specific to DST are important to include because they show specific DST-related properties of models, we think that it is also useful to include time series plots to help the reader conceptualize the model, to make judgments about the plausibility of the model, and, where both modeled and empirical data are available and plotted together, to make judgments about how well the model fits the data.

When results are presented from simulations in the absence of empirical data (e.g., Peluso et al., 2012), showing a few representative time series plots of the simulated data could help readers gain a better sense of how the patterns described in other ways (e.g., other plots, text descriptions) would look in empirical data. Readers might be able to get some sense of the plausibility of the model based on the look of these simulated time series plots. When empirical data have been collected (e.g., Cook et al., 1995; Gottman et al., 1999; Chow et al., 2005; Boker and Laurenceau, 2006; Fisher et al., 2011), presenting time series plots of these data along with time series plots generated from the models (on the same scale), would allow the reader to get a visual sense of how well the models fit. If different models are fit to the same data (e.g., Hufford et al., 2003; Witkiewitz et al., 2007), a time series for each model should be included. The time series plots should be generated at the same level (e.g., individual, group) for which the dynamical system variables are described in the model.

In our opinion, because time series plots do not require technical expertise for interpretation, showing empirical and modeled time series plots together is a way of presenting results that is particularly accessible to lay readers. While we did not encounter this kind of presentation in any of the articles relevant to clinical psychology that we looked at, an example from the biological sciences can be found in Figures 4B and 4D of Shiferaw et al. (2006). In these figures, an empirical time series plot of calcium transients in a stimulated rat cardiac muscle cell is shown alongside a corresponding time series plot generated from a non-linear dynamical model. Despite slight differences between the empirical and model plots, we believe that the overwhelming resemblance of the two plots provides a compelling illustration, even to readers with no knowledge of DST or cell biology, of the excellent match of the dynamical model to the empirical data. We believe that similar presentations in psychology articles would provide much clearer evidence than model fit statistics, or other statistical measures, of the value of dynamical models.

## Conclusion

Is DST a useful approach for clinical psychology research? Has it already made contributions to the field? We are not sure, and believe that it is impossible, at this point, for non-expert readers to determine.

We hope that by providing clearer information about the role of DST models in their work, and about the fit of their models to data, researchers applying DST to psychological variables will better enable the psychology research community to answer these questions.
